# Screening of Cyanide-Utilizing Bacteria from Rumen and In Vitro Evaluation of Fresh Cassava Root Utilization with Pellet Containing High Sulfur Diet

**DOI:** 10.3390/vetsci8010010

**Published:** 2021-01-15

**Authors:** Rittikeard Prachumchai, Anusorn Cherdthong, Metha Wanapat

**Affiliations:** Tropical Feed Resources Research and Development Center (TROFREC), Department of Animal Science, Faculty of Agriculture, Khon Kaen University, Khon Kaen 40002, Thailand; rittikeard1994@gmail.com (R.P.); metha@kku.ac.th (M.W.)

**Keywords:** thiocyanate, cyanide-utilizing bacteria, rumen fermentation, cyanide, degradability

## Abstract

Two experiments were undertaken to screen for ruminal cyanide-utilizing bacteria (Experiment 1), and to evaluate the influence of fresh cassava root (FCR) and pellets containing high sulfur (PELFUR) on cyanide content, gas production parameters, in vitro degradability, and ruminal fermentation (Experiment 2). Experiment 1 was conducted in a completely randomized design (CRD) for the screening of cyanide-utilizing bacteria and the dietary treatments consisted of cyanide at 0, 150, 300, and 450 ppm. In Experiment 2, a 5 × 3 factorial arrangement in a completely randomized design was used for the in vitro study. Factor A was the level of FCR at 0, 260, 350, 440, and 530 g/kg of dry matter (DM) substrate, and factor B was the level of PELFUR at 0, 15, and 30 g/kg DM substrate. In Experiment 1, adding different doses of cyanide significantly affected cyanide-utilizing rumen bacterial growth (*p* < 0.05). Increasing the concentration of cyanide from 0 to 150 and 150 to 300 ppm resulted in increases in cyanide-utilizing rumen bacteria of 38.2% and 15.0%, respectively. In Experiment 2, no interaction effects were found between FCR and PELFUR doses on gas production parameters (*p* > 0.05). Increasing the FCR level to more than 260 g/kg of DM substrate could increase cumulative gas production (*p* < 0.05). Increasing doses of PELFUR from 15 to 30 g/kg increased the cumulative gas production when compared with that of 0 g PELFUR/kg of DM substrate (*p* < 0.05). The cyanide concentration in rumen fluid decreased with PELFUR (*p* < 0.05) supplementation. Degradability of in vitro DM and organic matter following incubation increased at 12 and 24 h due to PELFUR supplementation with FCR and increased additionally with 15 g PELFUR/kg of DM substrate (*p* < 0.05) in 440 g FCR/kg of DM substrate. Proportions of the total volatile fatty acids, acetic acid (C2), propionic acid (C3), and butyric acid among supplementations with FCR (*p* < 0.05) were significantly different. In conclusion, the present results represent the first finding of bacteria in the rumen that are capable of utilizing cyanide, and suggests that cyanide might function as a nitrogen source for bacterial cell synthesis. The inclusion of FCR of 530 g/kg with 30 g PELFUR/kg of DM substrate could increase the cumulative gas production, the bacterial population, the in vitro degradability, the proportion of C3, and the rate of the disappearance of cyanide.

## 1. Introduction

An energy source, cassava root, is used for animal diets, especially in the tropical region [1]. It can be used for human food and as an energy ingredient in animal diets. Normally, cassava root is sliced and sun-dried, called cassava chip, prior to its use as an ingredient in animal diets. The dried form of the cassava root has been reported to have high degradability in the rumen (95–99%) [2]. Feeding cassava roots in their fresh form to ruminants is more feasible in all seasons [3]. Cherdthong et al. [2] stated that feeding fresh cassava root (FCR) could be another way to increase the use of cassava roots without prior processing. However, cyanogenic compounds contained in cassava roots, including linamarin and lotaustralin, are constraints for FCR usage. These compounds, cyanohydrins, are produced via a hydrolysis process, starting with linamarin as a precursor to linamarase. The more hydrolysis there is, the more toxic the root may be [4].

Cyanide poisoning is related to the amount of feed consumed and the animal’s physiological condition, and a cyanide level exceeding 200 mg/kg on a wet weight basis is dangerous. On a dry matter basis, feeds with more than 500 mg cyanide/kg should be considered potentially toxic [5]. Cherdthong et al. [2] demonstrated that an average cyanide content of 85 to 114 mg/kg on a fresh matter (FM) basis is contained in FCR. Promkot and Wanapat [6] suggested that microbes in the rumen could be able to eliminate the negative effects of cyanide. Similarly, Jones and Megarrity [7] demonstrated that *Leucaena* toxicity (mimosine) can be degraded by rumen microbe, namely, *Synergistes jonesii*. However, no previous report has elucidated whether the rumen microbes are able to degrade cyanide. Two enzymes are of particular interest in the rumen microbes—rhodanase and mercaptopyruvate sulfurtransferase. Rhodanese is a mitochondrial enzyme that functions to detoxify cyanide into thiocyanate, which is safe, then excrete it out of the ruminant via urine [8]. The conversion of thiocyanate from cyanide via rhodanese involves a cofactor, called sulfane sulfur. This cofactor’s availability relies on the availability of an amino acid having sulfur in its structure. In diets containing low amounts of these amino acids, the addition of sulfur is suggested [9]. Cherdthong et al. [2] found that supplementation of FCR at 10 g/kg and 15 g/kg of body weight (BW) with feed blocks containing sulfur at 20 and 40 g/kg did not have a negative effect on digestibility and rumen fermentation, and cyanide from FCR can be changed to thiocyanate, which is less toxic to animals. Additionally, Supapong et al. [10] suggested that 20 g sulfur/kg supplementation in fermented total mixed ration (FTMR) containing FCR and fermented for 7 days improved the digestibility of nutrients; microbial protein synthesis effectiveness; and the amount of total volatile fatty acids (VFAs), propionic acid, and thiocyanate in the blood. However, feeding animals with feed products such as sulfur-containing pellets requires, in particular, an increase in value-added feed and a convenient practical use for farmers.

Pelleted feeds have been successfully used for animals. Pelleting improves the palatability, density, and quality of feedstuff [11]. Pellet diets could enhance nutrient digestibility and fermentation in the rumen [12,13]. According to our review, a study on the influence of FCR and pellets containing sulfur on changes in ruminal fermentation has not yet been conducted. It was hypothesized that FCR and pellets containing sulfur could reduce cyanide content and enhance ruminal fermentation, mainly propionic acid production.

Therefore, the current work aimed to screen for ruminal cyanide-utilizing bacteria and evaluate the influence of FCR and pellets containing high sulfur (PELFUR) on cyanide content, gas production parameters, in vitro degradability, and ruminal fermentation.

## 2. Materials and Methods

### 2.1. Experiment 1

#### 2.1.1. Screening of Cyanide-Utilizing Bacteria in the Rumen

##### Animals and Feeding

Two donors, fistulated dairy cows 370 ± 10 kg in weight, were used. Cows were offered a concentrate composed of 140 g/kg DM crude protein (CP), 410 g/kg DM ether extract (EE), 220 g/kg DM neutral detergent fiber (NDF), 110 g/kg DM acid detergent fiber (ADF), and total digestible nutrient of 756 g/kg DM. Cows were fed at 5 g/kg of their weight daily for 21 days at 7:00 and 15:30 and rice straw was fed on an ad libitum basis. Free water and a mineral-block were provided for each cow housed individually. The rumen fluid (100 mL) was obtained from each cow before morning feeding, and then was filtered through layers of cheesecloth into warmed thermos bottles (39 °C) and transported to the laboratory using a sealed thermos under continuous flushing with carbon dioxide.

##### Sampling and Enrichment Culture for Cyanide-Utilizing Bacteria in Rumen

After obtaining inoculum, a 500-mL Erlenmeyer flask was filled with 90 mL of saline solution with 1 g/L Tween 80. The solution made moving microorganisms from the rumen fluid to the aqueous phase easier. Then, the saline solution was added to 10 mL of rumen fluid and mixed at 39 °C and 150 rpm for 1 h. The enrichment of the microorganism was done in a fresh medium by 100 g/L v/v inoculation every 4 days. Following three successive subcultures as above, 0.1 mL crop aliquots were streaked on nutrient agar plates with a nitrogen source of 1 mM cyanide. Nutrient agar plates were distilled for the isolation of colonies at 4 °C. The pH (6.5–7.0) was maintained with 0.1 NaOH, and 10 mL or 100 g/L of cultured H_2_SO_4_ was added to the flask to test for cyanide-utilizing bacteria [14].

##### Media and Culture Conditions

According to Kandasamy et al. [14], NaCN can be used as a sole nitrogen source for cyanide-utilizing bacteria in cassava factory wastewater. A mineral medium containing 10 g/L (*w*/*v*) NaCN and 10 g/L (*w*/*v*) glucose was the enrichment material. The mineral medium was composed of 0.1 M NaOH, 4.35 g/L K_2_HPO_4_, and 10 mL of a salt mixture containing 300 mg of ferrous sulfate heptahydrate, 180 mg of magnesium sulphate heptahydrate, 130 mg cobalt (II) chloride, 40 mg calcium chloride, 40 mg manganese (II) chloride tetrahydrate, and 20 mg molybdenum (VI) oxide in 1 L deionized water. The pH was balanced within a 6.5–7 range. The medium was sterilized by autoclave at 15 psi and 121 °C for 20 min. The experiment was conducted in a completely randomized design. Dietary treatments were the level of cyanide at 0, 150, 300, and 450 ppm. Cyanide as the NaCN solution (1 g/L) and glucose (1 g/L) were filter-sterilized before being added into the samples (0.2 mL pore size; Advantec Toyo Kaisha, Ltd., Tokyo, Japan). The NaCN was inserted via a silicone septum into the culture using a syringe [15].

##### Bacterial Growth

Microorganisms were counted using the plate counting method [16] and recorded as cfu/g fresh matter (FM). Ten grams of FM in 10^−1^ to 10^−5^ sodium chloride solution were applied to 90 mL of sterilized distilled water, shaken well by hand, and serially diluted by 8.5 g/L. For each dilution, 20 μL were placed on agar plates. In anaerobic conditions (Sugiyama-gen Co., Ltd., Tokyo, Japan), the cyanide-utilizing rumen bacteria were counted on agar media composed of peptone (0.5 g), beef extract (0.3 g), sodium chloride (0.8 g), and agar (15.0 g) after an incubation for 96 h at 39 °C.

### 2.2. Experiment 2

#### 2.2.1. In Vitro Study

##### Pellets Containing High Sulfur (PELFUR) Preparation and Experimental Design

Feedstuffs were milled through a 0.1-mm screen before being used as a substrate. Instead, all the ingredients were blended together well. The blended ingredients were pelleted and sun-dried for about 3 days to ensure suitable moisture content [12]. The fresh cassava root (FCR), Kasetsart 50 variety, was purchased from a local producer in Khon Kaen, Thailand. The age of harvesting FCR was 1 year, and they were washed, chopped, and checked immediately.

The work was conducted in a 5 × 3 factorial arrangement in a completely randomized design (CRD). Factor A was the level of FCR at 0, 260, 350, 440, and 530 g/kg of dry matter (DM) substrate, and factor B was the level of PELFUR at 0, 15, and 30 g/kg of DM substrate. The 0.5 g substrate contained a roughage-to-PELFUR ratio of 70:30, and the roughage diet was rice straw. The PELFUR, rice straw, and FCR samples were ground to pass through a 1-mm screen and perform the analysis of nutrient composition including CP, DM, and ash content [17]. Van Soest et al. [18] method was followed to analyze the content of ADF and NDF. Based on spectrophotometry, the content of cyanide in FCR was analyzed using the procedure of Bradbury et al. [19] and the obtained absorbance values were multiplied by the coefficient value of 396, expressed as mg/kg. Table 1 indicates the ingredients and nutrients of the PELFUR, rice straw, and FCR.

##### Inoculum

Two donors, fistulated dairy cows, 370 ± 10 kg in weight, were used. Cows were offered a concentrate, composed of 140 g/kg CP, 410 g/kg DM EE, 220 g/kg DM NDF, 110 g/kg DM ADF, and total digestible nutrients of 756 g/kg. Cows were fed 5 g/kg of their weight daily for 21 days at 7:00 and 15:30 h. Free access to water and a mineral block was equipped for each cow housed individually. Before morning feeding, 3500 mL of ruminal fluid were collected from animals and then filtered through layers of cheesecloth into warmed thermos bottles and transported to the laboratory using a sealed thermos under continuous flushing with carbon dioxide.

##### Substrate

Three hundred and fifty milligrams of rice straw and 150 mg of PELFUR (70:30, rice straw-to-PELFUR ratio) were weighed and added to 50-mL glass bottles, then FCR was added at respective levels on a DM basis of 0, 131, 175, 219, and 262 mg DM. The dietary treatments were tested in triplicate within the incubation, and incubations were repeated on three separate days (runs). Three blanks (no substrate) were always included with each run. The Menke and Steingass [20] procedure was used for the preparation of artificial saliva. A ratio of 2 mL rumen fluid and 1 mL artificial saliva was mixed, incubated under a temperature of 39 °C, and continuously flushed with carbon dioxide. Then, a 40-mL inoculum mixture was injected into the bottles, closed using stoppers made of rubber and caps made of aluminum, and warmed under 39 °C temperature. During the incubation, the gas production was collected at 0, 1, 2, 4, 6, 8, 10, 12, 18, 24, 48, 72, and 96 h.

##### Analysis of Samples

An incubation series as described in the procedure of Schofield [21] was performed for the recording of produced gas, and the data concerning the produced gas were loaded in the equation *V_t_* = *V_f_* × (1-exp*^(−k(t−L))^*), where *V_t_* is the volume of gas at time *t*, *V_f_* is the final asymptotic gas volume corresponding to complete substrate digestion (mL/g DM), *t* is the incubation time (h), *k* is a rate constant (units time^−1^), and *L* is a discrete lag term (h).

Incubation times of 2 and 4 h were used to measure the pH for each bottle. After measuring pH, 18 mL of inoculum was taken and kept in 2 mL H_2_SO_4_ for later analysis of the content of NH_3_-N and VFAs. The Kjeldahl procedure [17] was performed for analyzing the content of NH_3_-N, and the procedure of Samuel et al. [22] was used for analyzing the molar contents of VFAs including acetate (C2), propionate (C3), and butyrate (C4) by means of high-performance liquid chromatography (Nova-Pak C18 column, size 4 × 150 mm, Waters Corporation, Milford, MA, USA). The total VFAs was the sum of the molar contents of VFAs. Another 1 mL of inoculum and 6 mL of formalin were mixed to count the number of protozoa and bacteria [23]. Spectrophotometry was used to measure the cyanide concentration in the fermentation liquor [24].

Incubation times of 12 and 24 h were selected for the study of degradability. A sample from each replication of each treatment was transferred into a Gooch crucible (40 mm void fraction), and the weight was recorded. The residues in each bottle were washed with distilled water. The crucibles were then oven dried to measure the DM content. The data of DM content were subtracted from the DM content in blanks, and the g/kg of DM degradability (IVDMD) was calculated. After that, the crucibles were heated at 550 °C for 6 h. The ash data were used to calculate the organic matter (OM) content and g/kg of OM degradability (IVOMD) [25].

#### 2.2.2. Statistical Analysis

Cyanide-utilizing bacteria data were analyzed using the PROC GLM of SAS [26] in a completely randomized design. The model is:

*Y*_ij_ = *µ*+ *A*_i_ + *ε*_ij_

where *Y*_ij_ is the data of cyanide-utilizing bacteria, *µ* is the overall mean, *A*_i_ is the cyanide levels effect (i = 1–4), and *ε*_ij_ is the residue. Duncan’s multiple range test was run to check the statistical differences of treatment means at *p* < 0.05.

Data from the in vitro study were analyzed using the PROC GLM of SAS [26] according to a 5 × 3 factorial arrangement in a completely randomized system. The model is:

*Y*_ijk_ = *μ* + *a*_i_ + *b*_j_ + *ab*_ij_ + *ε*_ijk_

where *Y*_ijk_ are the response variance; *μ* is the overall mean; *a*_i_ is the level of FCR at 0, 260, 350, 440, and 530 g/kg of 0.5 g DM (i, 1–5); *b*_j_ is the level of PELFUR at 0, 15 and 30 g/kg of DM substrate (j, 1–3); *ab*_ij_ is the interaction effect; and *ε*_ijk_ is the residue. The means of response variances were reported with the standard error of the mean. Duncan’s multiple range test was run to check the statistical differences of treatment means at *p* < 0.05.

## 3. Results and Discussions

### 3.1. Screening of Cyanide-Utilizing Bacteria from Rumen

**Figure 1:**
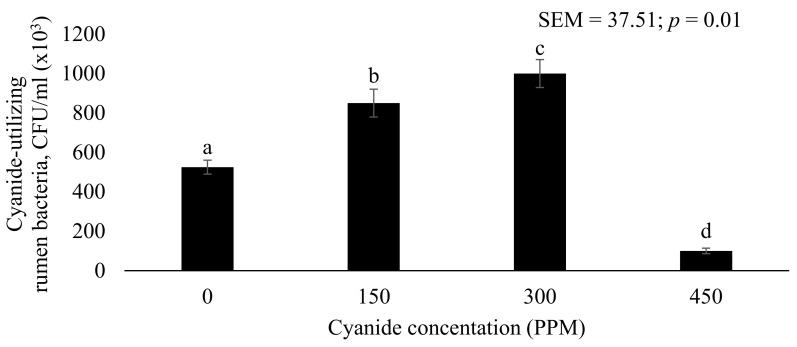
Cyanide-utilizing rumen bacteria growth curve at 0, 150, 300, and 450 ppm initial cyanide concentration.

Figure 1 shows the effect of cyanide concentration on the dynamic growth of cyanide-utilizing bacteria. Adding different doses of cyanide significantly affected cyanide-utilizing rumen bacterial growth (*p* < 0.05). The population of cyanide-utilizing bacteria were 525 × 10^3^, 850 × 10^3^, 1000 × 10^3^, and 100 × 10^3^ cfu/g FM for media containing cyanide at 0, 150, 300, and 450 ppm, respectively. Increasing the concentration of cyanide from 0 to 150 and 150 to 300 ppm resulted in an increase in cyanide-utilizing rumen bacteria of 38.2% and 15.0%, respectively. These results confirm the hypothesis that the rumen fluid contains bacteria which break down cyanide for the use of cell synthesis. Subsequently, it could be assumed that cyanide is used by the bacteria as a nutrient for their growth, with cyanide acting as a nitrogen source. This finding is consistent with a study by Razanamahandry et al. [27] which showed that certain bacteria are capable of using cyanide as a nitrogen source through enzymes which catalyzed the conversion of the sulfur species to rhodanese and mercaptopyruvate sulfurtransferase. Nevertheless, increasing the concentration of cyanide from 300 to 450 ppm decreased the amount of cyanide-utilizing rumen bacteria by 90%. This could be due to the addition of cyanide up to 450 ppm resulting in high bacterial toxicity and thus inhibiting cytochrome oxidase and interfering with some of the key biochemical reactions and activities (i.e., the reduction of oxygen in the cytochrome respiratory chain, the electron transport chain, and the activity of enzymes such as catalase and oxidase) [28]. The present results demonstrate that bacteria are able to detoxify a cyanide concentration not more than 450 ppm in 0.5 g substrate and can be applied to feeding animals with diets containing cyanide at a level lower than 450 ppm. This is in agreement with the findings of Kang and Kim [29], who studied the degradation of cyanide by a mixture of bacteria and demonstrated that cyanide played a role as a substrate up to 300 ppm, in which the mixture promoted the maximum growth rate of bacteria and the removal of cyanide. This is the first discovery of cyanide-utilizing bacteria in the rumen capable of degrading cyanide, and cyanide might function as a nitrogen source for bacterial cell synthesis. Furthermore, several reports demonstrate that antinutritive substances can be detoxified by specific microbes contained in the rumen. Jones and Megarrity [7] stated that the infusion of ruminants with a mixed bacterial culture introduced from Hawaiian goats could resolve the leucaena toxicity problem in northern Australia, as they contain *Synergistes jonesii*, capable of degrading mimosine, 3,4-dihydroxy pyridine (3,4 DHP), and 2,3 DHP. Similarly, Intanoo et al. [30] found that isolated rumen yeast could detoxify aflatoxin contained in the diet of dairy cattle. Thus, the degradation of cyanide in the present in vitro study indicates that rumen bacteria might solve the cyanide toxicity problem in ruminants when feedstuffs containing cyanide are used as feed. However, the investigation of the species of cyanide-utilizing bacteria in the rumen is required for future studies.

### 3.2. In Vitro Gas Production Study

#### 3.2.1. Gas Production Parameters and Cumulative Gas Production

**Figure 2:**
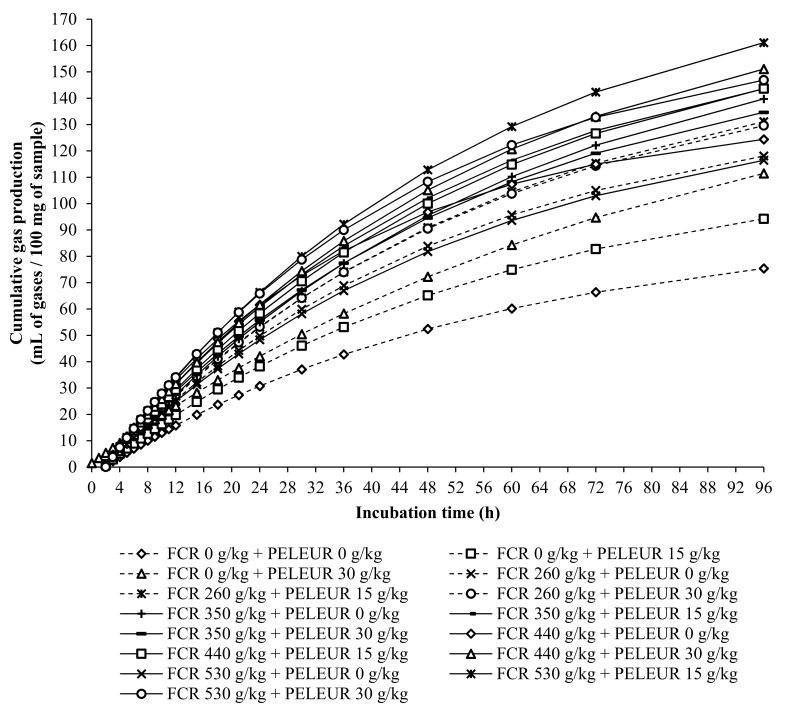
Effect of fresh cassava root (FCR) with pellets containing high sulfur (PELFUR) on cumulative gas during incubation times.

Figure 2 presents the cumulative gas production dynamics at various hours of incubation, and values of gas production parameters and cumulative gas production at 96 h are listed in Table 2. No interaction effects were found between FCR and PELFUR doses in terms of final asymptotic gas volume, corresponding to complete substrate digestion (*V_f_*), a rate constant (*k*), a discrete lag term (*L*), and cumulative gas production at 96 h of incubation (*p* > 0.05). However, increasing the FCR in the diet (0 to 530 g/kg) affected the final asymptotic gas volume, corresponding to complete substrate digestion, a discrete lag term, and cumulative gas production at 96 h of incubation (*p* < 0.05), which ranged from 91.85 to 193.70 mL, 0.50 to 2.10 h, and 75.43 to 161.09 mL, respectively. Increasing the FCR level to more than 260 g/kg of DM substrate was able to increase cumulative gas production, whereas increasing the PELFUR from 0 to 30 g/kg affected the final asymptotic gas volume, corresponding to complete substrate digestion (*p* < 0.05) but had no effect on a discrete lag term (*p* > 0.05). These results demonstrated that the inclusion of FCR and PELFUR improves in vitro fermentation by rumen microbes [21]. Moreover, increasing doses of PELFUR from 15 to 30 g/kg increased the cumulative gas production when compared with that of 0 g PELFUR/kg. This might be due to the increased number of microorganisms [4], which could improve feed degradability and enhance the performance of cumulative gas production. Furthermore, FCR is filled with starch; thus, carbohydrates are shifted from cassava to starch, leading to higher nutrient availability and resulting in effective cumulative gas production [3].

#### 3.2.2. In Vitro Fermentation and Ruminal Microbial Population

Table 3 presents the effect of FCR supplementation with PELFUR on in vitro rumen fermentation and the ruminal microbial population using an in vitro gas development test. There was no interaction between FCR and PELFUR levels on all the parameters (*p* > 0.05). Increasing doses of FCR supplementation decreased ruminal pH. This could be due to an increase in FCR containing fermentable carbohydrate, which is rapidly fermented, resulting in decreased pH. The pH continued dropping, averaging 6.90–6.86, possibly due to the bioconversion of available carbohydrates. However, the ruminal pH for FCR and PELFUR was within the appropriate range for microbial synthesis and feed fermentation activity in rumen microbial enzyme degradation and feed fermentation activity in the rumen [31].

Concentrations of NH_3_-N were significantly impacted (*p* < 0.05) and decreased with increased rates of FCR supplementation. This could be due to a sufficient supply of energy from a rapidly fermentable source of carbohydrates to allow the efficient incorporation of peptides, free amino acids, and NH_3_-N into microbial cells [10]. Likewise, Cherdthong et al. [2] found that the concentration of NH_3_-N in the rumen increased with an increasing amount of FCR supplementation. Increasing the amount of PELFUR by 15 and 30 g/kg also reduced the concentration of NH_3_-N by 2.44 and 2.42 mg/dL compared to the control PELFUR group. This might be due to ruminal microorganisms that use NH_3_-N as a non-protein nitrogen source substrate for cell synthesis. Thus, microbial protein synthesis might require the use of NH_3_-N with sulfide [3]. This result is consistent with what Supapong et al. [10] found, that ruminal NH_3_-N concentrations were slightly lower (15.20 mg/dL) and complemented well with sulfur at 20 g/kg. Additionally, the FCR supplementation and PELFUR did not affect the protozoal and bacterial populations (*p* > 0.05), ranging from 5.00 × 10^6^ to 9.00 × 10^6^ cells/mL and 9.50 × 10^8^ to 13.00×10^8^ cells/mL, respectively.

The cyanide concentration in rumen fluid, as affected by PELFUR supplementation with FCR, is presented in Table 3. The results show that the cyanide concentration in the rumen fluid decreased with PELFUR (*p* < 0.05) supplementation. The doses of 15 and 30 g PELFUR/kg decreased the concentration of cyanide by 30.35–36.96% and 37.06–40.08%, as compared to the control. The reduction of cyanide in the substrate could be due to the influence of the addition of sulfur. The availability of sulfur plays an important role in the degradation mechanism of microorganisms. Rhodanese can extract cyanide from cyanide-utilizing rumen bacteria. Sulfur transferase rhodanese reduces thiocyanate toxicity by lowering the content of cyanide and thiosulphate. Supapong et al. [10] indicated that the theoretical reason for 1.2 g of sulfur to transfer to thiocyanate is to replace 1 g of cyanide. This is in line with the study of Cherdthong et al. [2], which revealed that the addition of 20 g sulfur/kg and 40 g sulfur/kg into the feed block to beef cattle receiving FCR can ensure sufficient cyanide reduction and conversion of thiocyanate. Furthermore, the above experiment of the screening of cyanide-utilizing rumen bacteria confirmed that increasing the dose of cyanide from FCR might stimulate these bacteria to detoxify cyanide. Therefore, providing sufficient substrates for the cell synthesis of cyanide-utilizing rumen bacteria, such as energy, amino acid, and nitrogen sources, could enhance bacterial growth and reduce cyanide concentrations.

#### 3.2.3. In Vitro Degradability

Table 4 provides the results concerning in vitro degradability at different incubation periods. Effects of the interaction between FCR and PELFUR on degradability were observed (*p* < 0.05). The results showed that the in vitro degradability of dry matter (IVDMD) and the in vitro degradability of organic matter (IVOMD) following incubation increased at 12 and 24 h due to PELFUR supplementation with FCR and increased more (651.7 g/kg DM and 672.1 g/kg DM; 690.0 g/kg DM and 725.9 g/kg DM, respectively) with 15 g PELFUR/kg (*p* < 0.05) in 440 g FCR/kg. This is probably due to the high concentration of nonstructural carbohydrates in the FCR, which can easily be broken down in the rumen and increase the bacterial population by improving the degradability of DM and OM in vitro [3]. Sulfur is also an important part of the rumen bacteria. The ruminant relies on ruminant microbes to transform sulfate into hydrogen sulfite, which is used to produce essential amino acids (cysteine and methionine) for the synthesis of microbial ruminants [6]. Consequently, an adequate supply of sulfur would enhance the growth of microorganisms and enhance nutrient digestion. Similar findings were reported by Cherdthong et al. [2], who revealed that cattle fed 40 g sulfur/kg had improved digestibility of DM and OM compared to those fed 20 g sulfur/kg in the feed block with supplementation of FCR at 10 and 15 g/kg of BW.

#### 3.2.4. Concentration of Volatile Fatty Acids (VFAs)

For total VFAs, VFA profiles, and the ratio of C2 to C3, no interactions were found between FCR and PELFUR (Table 5; *p* > 0.05). However, the proportions of the total VFAs, C2, C3, and C4, as well as the ratio of C2 to C3 between supplementations with FCR (*p* < 0.05) were significantly different. As the proportion of FCR increased to 530 g/kg of the DM substrate, the volume of C3 increased by 14.6%. These findings could be attributed to an increase in fermentable carbohydrate-containing FCR, which is rapidly fermented, thus resulting in high overall concentrations of VFA and C3 in the rumen. Essentially, high C3 is beneficial for animals as the principal source of the synthesis of live glucose through gluconeogenesis. These findings are similar to those of Cherdthong et al. [2], which showed that a high-FCR diet in Thai native cattle enhanced the C3 concentration, as compared with low FCR diets. Dagaew et al. [3] suggested that the change in the ratio of FCR to rice straw was 100:0, with 20 and 40 g sulfur/kg in the high sulfur feed block (FBS), enhancing the C3 ratio by 16.7% compared with the control group. Additionally, the sulfur concentration in PELFUR influenced the concentration of C3. When a high concentration of sulfur is added, the rise in the ruminal C3 concentration can imply that the C3 concentration can be used as a hydrogen sink when an excess of available ruminal sulfide is provided [10]. This is consistent with the findings of Promkot et al. [4], who revealed an increase in C3 in fresh cassava foliage with 10 g sulfur/kg supplementation, as sulfur was a precursor to microbial protein synthesis in rumen. Similarly, Supapong and Cherdthong [8] stated that supplementation of 20 g sulfur/kg FTMR containing FCR increased the propionic acid by 10.9%, as compared with the non-supplementation with sulfur.

## 4. Conclusions

The cyanide-utilizing bacteria in the rumen discussed in this study are the first to be found capable of degrading cyanide and withstanding cyanide at a quantity of 300 ppm in the media. However, the population decreased when a concentration of more than 450 ppm was employed. It could be concluded that the inclusion of FCR in 530 g/kg of DM substrate with 30 g PELFUR/kg could increase the cumulative gas production, the bacterial populations, the in vitro degradability, the proportion of propionic acid, and the rate of the disappearance of cyanide without having any adverse effect on in vitro fermentation. Nevertheless, in vivo studies are required to investigate the effects of FCR and PELFUR in practical feeding regimes.

## Figures and Tables

**Table 1:** Ingredient and chemical composition of pellets containing high sulfur (PELFUR) in the experiment (g/kg dry matter, DM).

Item	PELFUR 0	PELFUR 15	PELFUR 30	FCR	Rice Straw
Ingredients, g/kg DM					
Cassava chips	556	556	556	-	
Soybean meal	110	110	110	-	
Rice bran	110	100	105	-	
Coconut meal	89	89	70	-	
Palm kernel meal	78	73	72	-	
Urea	10	10	10	-	
Salt	10	10	10	-	
sulfur powder	0	15	30	-	
Mineral premix	10	10	10	-	
Molasses, liquid	27	27	27	-	
Chemical composition					
DM, g/kg as basis	946	943	941	351	925
Organic matter, g/kg DM	925	921	932	924	895
Ash, g/kg DM	75	79	68	76	105
Crude protein, g/kg DM	132	130	130	21	23
Neutral detergent fiber, g/kg DM	228	231	226	156	712
Acid detergent fiber, g/kg DM	104	106	104	89	442
Cyanide, ppm	-	-	-	106	-

FCR = fresh cassava root, PELFUR = pellets containing high sulfur.

**Table 2:** Effect of fresh cassava root (FCR) with pellets containing high sulfur (PELFUR) on gas production parameters and cumulative gas at 96 h after incubation.

Item	FCR (g/kg)	PELFUR (g/kg)	Gas Production Parameters			Cumulative Gas (mL)
			*V_f_*	*k*	*L*	
	0	0	91.85	0.02	1.60	75.43
	0	15	115.80	0.02	1.45	94.16
	0	30	153.95	0.02	0.50	111.40
	260	0	139.30	0.02	1.95	114.82
	260	15	159.75	0.02	1.90	131.04
	260	30	156.75	0.02	1.60	129.49
	350	0	173.65	0.02	2.10	139.68
	350	15	169.50	0.02	1.70	142.94
	350	30	161.40	0.02	1.75	133.71
	440	0	134.45	0.03	1.70	124.40
	440	15	173.65	0.02	2.10	143.45
	440	30	183.50	0.02	1.75	150.62
	530	0	139.75	0.02	1.55	116.05
	530	15	193.70	0.02	1.80	161.09
	530	30	166.05	0.02	2.00	146.27
SEM			13.19	0.01	0.95	9.08
Main effects						
FCR (g/kg)	0		120.53 ^a^	0.02	1.18 ^a^	93.66 ^a^
	260		151.93 ^b^	0.02	1.82 ^b^	125.12 ^b^
	350		168.18 ^b^	0.02	1.85 ^b^	138.78 ^bc^
	440		163.87 ^b^	0.02	1.85 ^b^	139.49 ^bc^
	530		166.50 ^b^	0.02	1.78 ^b^	141.14 ^c^
PELFUR (g/kg)	0		135.80 ^a^	0.02	1.78	114.07 ^a^
	15		162.48 ^b^	0.02	1.79	134.53 ^b^
	30		164.33 ^b^	0.02	1.52	134.30 ^b^
Significance of main effect and interaction						
FCR			0.01	0.64	0.01	0.01
PELFUR			0.01	0.82	0.16	0.01
FCR × PELFUR			0.15	0.99	0.15	0.29

^a,b,c^ Mean values in the same column with different superscripts are statistically different (*p* < 0.05), FCR = fresh cassava root, PELFUR = pellets containing high sulfur, SEM = standard error of the mean, *V_f_* = final asymptotic gas volume corresponding to complete substrate digestion (mL/500 mg DM), *k* = a rate constant (units time^−1^), *L* = a discrete lag term (h).

**Table 3:** Effect of fresh cassava root (FCR) with pellets containing high sulfur (PELFUR) on ruminal pH, ammonia-nitrogen (NH_3_-N) concentration, ruminal microbial population, and cyanide concentration.

Item	FCR (g/kg)	PELFUR (g/kg)	pH	NH_3_-N (mg/dL)	Protozoa (×10^6^ cell/mL)	Bacteria (×10^8^ cell/mL)	Cyanide (ppm)
	0	0	6.91	16.86	7.00	9.50	0.00 ^a^
	0	15	6.89	14.96	6.00	11.00	0.00 ^a^
	0	30	6.90	15.21	6.00	11.50	0.00 ^a^
	260	0	6.89	15.71	6.00	10.50	0.26 ^f^
	260	15	6.86	11.51	6.00	11.50	0.18 ^c^
	260	30	6.87	12.91	5.00	12.00	0.15 ^b^
	350	0	6.87	14.31	7.00	11.00	0.34 ^k^
	350	15	6.86	12.21	7.00	12.00	0.24 ^e^
	350	30	6.85	12.21	7.00	12.50	0.21 ^d^
	440	0	6.85	15.71	6.00	11.50	0.43 ^l^
	440	15	6.83	12.96	9.00	12.50	0.29 ^h^
	440	30	6.83	12.41	8.00	12.50	0.27 ^g^
	530	0	6.82	17.26	8.50	12.00	0.51 ^m^
	530	15	6.83	16.01	8.00	13.00	0.32 ^j^
	530	30	6.83	15.01	9.00	13.00	0.31 ^i^
SEM			0.01	0.53	1.85	0.73	0.003
Main effects							
FCR (g/kg)	0		6.90 ^a^	15.68 ^a^	6.30	10.70	0.00 ^a^
	260		6.87 ^b^	13.38 ^b^	5.70	11.30	0.20 ^b^
	350		6.86 ^c^	12.91 ^b^	7.00	11.80	0.26 ^c^
	440		6.84 ^d^	13.69 ^b^	7.70	12.20	0.33 ^d^
	530		6.83 ^d^	16.09 ^a^	8.50	12.70	0.38 ^e^
PELFUR (g/kg)	0		6.87 ^a^	15.97 ^a^	6.90	10.90	0.31 ^a^
	15		6.85 ^b^	13.53 ^b^	7.20	12.00	0.21 ^b^
	30		6.86 ^b^	13.55 ^b^	7.00	12.30	0.19 ^c^
Significance of main effect and interaction							
FCR			0.01	0.01	0.40	0.22	0.01
PELFUR			0.04	0.01	0.97	0.65	0.01
FCR × PELFUR			0.28	0.23	0.98	0.90	0.01

^a–j^ Mean values in the same column with different superscripts are statistically different (*p* < 0.05), FCR = fresh cassava root, PELFUR = pellets containing high sulfur, SEM = standard error of the mean.

**Table 4:** The effect of fresh cassava root (FCR) with pellets containing high sulfur (PELFUR) on the in vitro digestibility of nutrients.

Item	FCR (g/kg)	PELFUR (g/kg)	IVDMD (g/kg DM)			IVOMD (g/kg DM)		
			12 h	24 h	Mean	12 h	24 h	Mean
	0	0	562.6 ^a^	598.8 ^a^	580.7 ^a^	600.8 ^a^	639.5 ^a^	620.2 ^a^
	0	15	569.5 ^a^	603.7 ^ab^	586.6 ^a^	612.7 ^ab^	647.3 ^ab^	630.0 ^ab^
	0	30	559.6 ^a^	613.9 ^bc^	586.7 ^a^	601.6 ^a^	652.2 ^bc^	626.9 ^ab^
	260	0	582.9 ^c^	611.7 ^abc^	597.3 ^b^	623.1 ^bc^	652.6 ^bc^	637.8 ^bc^
	260	15	601.7 ^dh^	621.7 ^c^	611.7 ^c^	644.6 ^de^	663.5 ^c^	654.1 ^de^
	260	30	597.2 ^d^	637.2 ^de^	617.2 ^cd^	637.9 ^d^	679.0 ^d^	658.5 ^ef^
	350	0	603.9 ^bdh^	616.2 ^bc^	610.1 ^c^	630.5 ^cd^	663.1 ^c^	646.8 ^cd^
	350	15	616.4 ^e^	649.3 ^ef^	632.9 ^e^	653.2 ^e^	689.6 ^de^	671.4 ^g^
	350	30	608.6 ^beh^	654.7 ^fg^	631.7 ^e^	644.0 ^de^	693.2 ^e^	668.6 ^fg^
	440	0	612.3 ^be^	635.7 ^d^	624.0 ^de^	637.8 ^d^	681.2 ^de^	659.5 ^ef^
	440	15	647.8 ^g^	668.5 ^h^	658.1 ^gh^	684.3 ^gh^	720.7 ^fg^	702.5 ^ij^
	440	30	629.0 ^f^	664.2 ^gh^	646.6 ^f^	668.4 ^f^	713.0 ^f^	690.7 ^h^
	530	0	617.5 ^e^	643.5 ^def^	630.5 ^e^	642.2 ^de^	689.3 ^de^	665.7 ^fg^
	530	15	651.7 ^g^	672.1 ^h^	661.9 ^h^	690.0 ^h^	725.9 ^g^	708.0 ^j^
	530	30	633.7 ^f^	665.9 ^gh^	649.8 ^fg^	672.4 ^fg^	720.4 ^fg^	696.4 ^hi^
SEM			3.4	4.3	3.1	4.9	4.1	3.7
Main effects								
FCR (g/kg)	0		563.9 ^a^	605.4 ^a^	584.7 ^a^	605.0 ^a^	646.3 ^a^	625.7 ^a^
	260		593.9 ^b^	623.5 ^b^	608.7 ^b^	635.2 ^b^	665.0 ^b^	650.1 ^b^
	350		609.6 ^c^	640.1 ^c^	624.9 ^c^	642.6 ^c^	682.0 ^c^	662.3 ^c^
	440		629.7 ^d^	656.1 ^d^	642.9 ^d^	663.5 ^d^	705.0 ^d^	684.2 ^d^
	530		634.3 ^d^	660.5 ^d^	647.4 ^d^	668.2 ^d^	711.9 ^d^	690.0 ^d^
PELFUR (g/kg)	0		605.6 ^a^	621.2 ^a^	608.5 ^a^	626.9 ^a^	665.1 ^a^	646.0 ^a^
	15		617.4 ^b^	643.0 ^b^	630.2 ^b^	657.0 ^b^	689.4 ^b^	673.2 ^b^
	30		595.8 ^c^	647.2 ^b^	626.4 ^b^	644.9 ^c^	691.6 ^b^	668.2 ^b^
Significance of main effect and interaction								
FCR			0.01	0.01	0.01	0.01	0.01	0.01
PELFUR			0.01	0.01	0.01	0.01	0.01	0.01
FCR × PELFUR			0.01	0.01	0.01	0.01	0.01	0.01

^a–j^ Mean values in the same column with different superscripts are statistically different (*p* < 0.05), FCR = fresh cassava root, PELFUR = pellets containing high sulfur, SEM = standard error of the mean, IVDMD = in vitro dry matter digestibility, IVOMD = in vitro organic matter digestibility.

**Table 5:** The effect of fresh cassava root (FCR) with pellets containing high sulfur (PELFUR) on volatile fatty acid (VFA) concentrations.

Item	FCR (g/kg)	PELFUR (g/kg)	Total VFAs (mmol/L)	Acetate C2	Propionate C3	Butyrate C4	C2:C3
				(mol/100 mol)			
	0	0	91.40	67.26	23.44	9.30	2.87
	0	15	91.60	67.41	23.37	9.22	2.89
	0	30	92.24	66.91	23.61	9.49	2.83
	260	0	93.07	66.19	24.82	8.98	2.67
	260	15	93.54	65.41	25.47	9.12	2.57
	260	30	94.83	65.06	25.49	9.45	2.55
	350	0	95.13	64.99	25.18	9.84	2.58
	350	15	98.08	64.78	25.70	9.52	2.52
	350	30	96.15	64.71	25.52	9.76	2.54
	440	0	99.42	64.56	25.64	9.81	2.52
	440	15	100.71	63.53	27.01	9.46	2.35
	440	30	103.88	63.89	26.67	9.43	2.40
	530	0	106.45	63.86	26.93	9.21	2.37
	530	15	108.82	63.48	28.27	8.25	2.25
	530	30	107.35	64.16	27.32	8.52	2.35
SEM			2.53	0.33	0.34	0.32	0.04
Main effects							
FCR (g/kg)	0		91.75 ^a^	67.19 ^a^	23.47 ^a^	9.33 ^b^	2.86 ^a^
	260		93.81 ^ab^	65.55 ^b^	25.26 ^b^	9.18 ^ab^	2.60 ^b^
	350		96.45 ^b^	64.83 ^c^	25.47 ^b^	9.71 ^b^	2.55 ^b^
	440		101.34 ^c^	63.99 ^d^	26.44 ^c^	9.57 ^b^	2.42 ^c^
	530		107.54 ^d^	63.83 ^d^	27.51 ^d^	8.66 ^a^	2.32 ^d^
PELFUR (g/kg)	0		97.09	65.37	25.20 ^a^	9.43	2.60 ^a^
	15		98.55	64.92	25.97 ^b^	9.11	2.51 ^b^
	30		98.89	64.95	25.72 ^b^	9.33	2.53 ^b^
Significance of main effect and interaction							
FCR			0.01	0.01	0.01	0.01	0.01
PELFUR			0.51	0.09	0.01	0.32	0.01
FCR × PELFUR			0.98	0.41	0.48	0.74	0.45

^a–d^ Mean values in the same column with different superscripts are statistically different (*p* < 0.05), FCR = fresh cassava root, PELFUR = pellets containing high sulfur, SEM = standard error of the mean.

## Data Availability

Not applicable.
